# Delirium in COVID-19: psychopharmacology considerations

**DOI:** 10.1192/j.eurpsy.2021.734

**Published:** 2021-08-13

**Authors:** M. Lemos, J. Rema, T. Reynolds De Sousa

**Affiliations:** Psychiatry, Centro Hospitalar Lisboa Norte, Lisboa, Portugal

**Keywords:** delirium, Psychopharmacology, COVID-19, neurological disorders

## Abstract

**Introduction:**

Delirium is characterized by fluctuating disturbance of consciousness, inattention, reduced awareness, hallucinations or delusions, occurring in 20% of hospital admissions. Central nervous system symptoms are the main form of neurologic injury in patients with COVID-19 and a significant portion of these patients presents with delirium. COVID-19 infection’s course and symptoms, as well as patient comorbidities can facilitate its onset, which is exacerbated by the frequent need for higher doses of sedation to suppress severe cough.

**Objectives:**

To summarize the most recent practices for management of delirium in COVID-19 infected patients, with emphasis on the psychopharmacology approach.

**Methods:**

Selective literature review via PubMed search, using the terms “delirium, neurological disorders, psychopharmacology and COVID-19”.

**Results:**

COVID-19 associated delirium can be presented in its hyperactive type with exuberant agitation, but also with additional clinical features such as rigidity, akinetic mutism, abulia and alogia. Psychopharmacological approaches may be needed for patients with agitation when there’s intractable stress or risk to self or others. In this group of patients, melatonin, alfa-2 agonists and low potency antipsychotics have been used as first line treatment. Trazodone, valproate, dopamine agonists, amantadine can be used. Other approaches such as correction of vitamin deficiencies and remdesivir can also play a role.

**Conclusions:**

Delirium remains frequently unrecognized. In the pandemic context of COVID-19 it is important to consider this infection as a cause of delirium and mind the misdiagnosis as a psychiatric condition. One should look for atypical features and be more thoughtful about the psychopharmacological approach.

